# A risk prediction model for medication safety: assessing cardiopulmonary adverse outcomes in 11,252 patients treated with ulinastatin

**DOI:** 10.3389/fphar.2026.1746148

**Published:** 2026-01-21

**Authors:** Rutong Hua, Ying Zhang, Jianxiong Deng, Qiqi Wen, Guozheng Li, Rende Fang, Zhuoyu Chen, Yuxuan Cao, Xi-Yong Yu, Jin Li, Zhongxiao Lin

**Affiliations:** 1 Guangzhou Municipal and Guangdong Provincial Key Laboratory of Molecular Target and Clinical Pharmacology, The NMPA and State Key Laboratory of Respiratory Disease, School of Pharmaceutical Sciences, Guangzhou Medical University, Guangzhou, China; 2 Guangdong Province Pharmacological Society, Guangzhou, Guangdong, China; 3 Guangzhou United Family Hospital, Guangzhou, China

**Keywords:** adverse cardiopulmonary outcomes, phase IV study, post-marketing reevaluation, protease inhibitor, ulinastatin

## Abstract

**Background:**

Ensuring medication safety is a critical public health issue, particularly for widely used drugs like ulinastatin in critical care. Proactively identifying patients at high risk for adverse drug events is key to promoting safer medication practices and improving patient outcomes. This study focuses on developing a practical tool to stratify the risk of cardiopulmonary adverse outcomes associated with ulinastatin use.

**Methods:**

A multicenter, retrospective cohort study was conducted using data from 11,252 patients treated with ulinastatin between 2014 and 2017, 34 were excluded from the final statistical analysis due to missing critical data. Consequently, the cohort for all subsequent analyzes comprised 11,218 patients. The outcome of interest was the occurrence of a cardiopulmonary adverse event. We employed logistic regression to identify independent clinical risk factors and used these to construct a simple, points-based risk scoring system. The model’s performance in discriminating between high-risk and low-risk patients was evaluated using the area under the receiver operating characteristic curve (AUC).

**Results:**

Among the cohort, 152 (1.35%) patients experienced a cardiopulmonary adverse outcome. Four factors were identified as independent predictors and incorporated into the risk model: low Ulinastatin dosage <300,000 U (OR (odds ratios) = 5.570, 95% CI (confidence intervals): 3.670–8.454, p < 0.001), duration of medication >1 day (OR = 2.165, 95% CI: 1.480–3.166, p < 0.001), concomitant medications (OR = 2.088, 95% CI: 1.414–3.083, p < 0.001), and treatment in the Intensive Care Unit (ICU) (OR = 3.737, 95% CI: 2.487–5.615, p < 0.001). The composite risk score demonstrated good predictive accuracy, with an AUC of 0.779 (95% CI: 0.741–0.817), significantly outperforming any single predictor.

**Conclusion:**

We developed and validated a simple, clinically actionable risk stratification tool for cardiopulmonary adverse events in patients receiving ulinastatin. This model can help clinicians and healthcare systems identify high-risk individuals before treatment initiation, facilitating targeted monitoring, informed decision-making, and personalized dosing strategies. The implementation of such a tool represents a tangible step towards enhancing medication safety protocols and promoting safer prescribing behaviors in clinical practice.

**Clinical Trial Registration:**

https://www.chictr.org.cn/showproj.html?proj=11439.

## 1 Introduction

Medication safety represents a cornerstone of public health and health promotion, particularly for drugs extensively utilized in critical care settings (Yao et al., 2020). Ulinastatin, a broad-spectrum protease inhibitor, is one such agent widely employed to manage inflammatory conditions including pancreatitis, severe pneumonia, and septic shock, and to mitigate inflammatory responses during major surgeries such as cardiopulmonary bypass (He et al., 2014; Liu et al., 2020). As an adjunctive therapy, it has been shown to effectively reduce hospital length of stay, improve survival rates, and enhance prognostic outcomes (Chen et al., 2017; Teng et al., 2025). While its pharmacological profile, primarily mediated through the inhibition of hydrolase activity and modulation of pro-inflammatory cytokines like TNF-α, IL-6, and IL-8, is well-recognized (Fukushima et al., 2021; Lagoo et al., 2018), the focus within public health must extend beyond efficacy to encompass the proactive management of associated risks.

The cardiopulmonary system, fundamental to human health and a primary target in systemic inflammatory responses, is particularly vulnerable (Raghuveer et al., 2020). Adverse outcomes affecting this system not only threaten survival but also profoundly impair long-term quality of life and functional capacity, placing a significant burden on healthcare systems and patients alike (Plesner et al., 2017). Although ulinastatin is generally considered protective towards cardiac and pulmonary tissues (He et al., 2015), its real-world safety profile necessitates rigorous, large-scale investigation. Clinical reports and pharmacovigilance data, such as that from the WHO (world health organization)’s VigiAccess database (Liang et al., 2024), confirm that cardiopulmonary adverse events, including atrial flutter, flushing, acute respiratory distress syndrome, and interstitial lung disease, constitute a notable proportion of reported issues associated with its use (Table 1), such as, cardiovascular disorders accounted for 8% of all adverse reactions associated with ulinastatin, with atrial flutter being the most frequently reported (14 cases). Vascular disorders constituted 4% of adverse events, among which flushing was the most common (7 cases). Similarly, respiratory disorders events, such as acute respiratory distress syndrome, interstitial lung disease, were also observed in previous observations. This evidence underscores that even therapeutic agents with protective intentions can contribute to patient harm if risk factors are not adequately identified and managed.

**TABLE 1:** Selected cardiovascular adverse events and Respiratory adverse events reported for ulinastatin from VigiAccess.

ADR	Number
Cardiac disorders	​
Cardiac flutter	14
Palpitations	11
Tachycardia	10
Bradycardia	6
Cardiac arrest	4
Arrhythmia	1
Cardiac failure acute	1
Cardiac tamponade	1
Pulseless electrical activity	1
Ventricular fibrillation	1
Ventricular tachycardia	1
Vascular disorders	​
Flushing	7
Hypotension	4
Shock	4
Vascular pain	2
Aneurysm ruptured	1
Circulatory collapse	1
Hyperaemia	1
Hypovolaemic shock	1
Phlebitis	1
Thrombophlebitis	1
Vasculitis	1
Respiratory disorders	​
Acute respiratory distress syndrome	1
Interstitial lung disease	1
Pneumothorax	1
Respiratory distress	1
Respiratory failure	1

Data from 1 Jan 1987, to 22 Aug 2025.

Despite its widespread use, significant challenges remain in clinical practice regarding inappropriate administration and its impact on cardiopulmonary recovery, complication prevention, and specific patient populations. Improper use of ulinastatin may lead to adverse cardiopulmonary outcomes, which not only substantially impair patients’ quality of life but also pose serious threats to survival. This highlights a critical gap between the drug’s therapeutic potential and the optimization of its safe application. A key challenge in promoting safer medication use, therefore, lies in the transition from passive adverse event reporting to active, pre-emptive risk stratification. Current practice often lacks simple, actionable tools to identify individuals at heightened risk prior to treatment initiation. Factors such as inappropriate dosing, complex medication regimens, and patient care settings (e.g., the ICU) are potential modifiers of risk, yet their collective impact remains poorly quantified in routine practice.

Therefore, moving from a reactive to a preventive paradigm is essential. This study leverages a large, multicenter Phase IV clinical trial dataset to address this public health need. We aim to identify key clinical risk factors for cardiopulmonary adverse outcomes following ulinastatin administration and, crucially, to integrate these factors into a validated, practical risk prediction model. The ultimate goal is to provide a decision-support tool that can guide clinicians in risk assessment before treatment, thereby fostering safer prescribing behaviors, enabling tailored patient monitoring, and contributing to the broader objective of optimizing medication safety outcomes in vulnerable populations.

## 2 Methods

### 2.1 Data sources

This multicenter, retrospective study analyzed post-marketing data from patients treated with ulinastatin (manufactured by Guangdong Techpool Bio-pharma Co., Ltd., China) between August 2014 and June 2017 (Li et al., 2022). The study cohort comprised patients admitted to general wards and ICUs across nine participating hospitals in China. The study protocol received approval from the Ethics Committee of Guangdong Provincial Hospital of Chinese Medicine (Approval No. B2014-056-01), which served as the lead center. All procedures were conducted in compliance with relevant guidelines and regulations. The Ethics Committee of Guangdong Provincial Hospital of Chinese Medicine granted a waiver for the requirement of informed consent.

### 2.2 Study design

**FIGURE 1:**
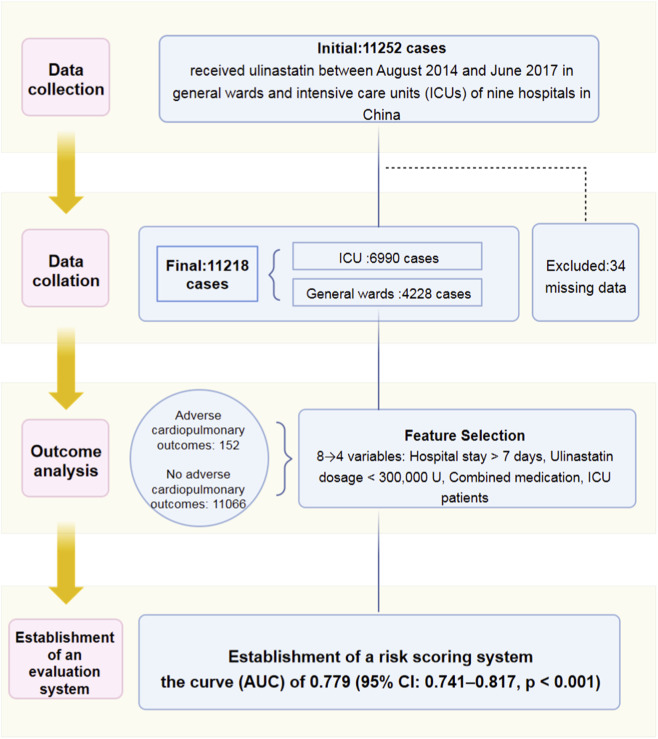
Patient selection flowchart and study overview. Of the 11,252 initially identified patients, 11,218 were included in the final analysis. Subsequently, 152 patients experienced cardiopulmonary adverse outcomes and were used to identify risk factors and develop a risk scoring system.

Excluded patients with missing data, this study enrolled 11,218 patients and the following data were collected: ulinastatin dosage (in 1 × 10^4^ U units), history of cardiopulmonary diseases, ICU admission status, length of hospital stay, gender, concomitant medications, allergy history, and occurrence of cardiopulmonary adverse outcomes. Based on the occurrence of such outcomes, the patients were categorized into two groups: those with cardiopulmonary adverse outcomes and those without. The cardiopulmonary adverse outcomes were ascertained from the Phase IV clinical trial database, to ensure consistency and accuracy in assessment, the terminology for all adverse outcomes was described in accordance with the Common Terminology Criteria for Adverse Events version 4.0 (CTCAE v4.0). All outcomes were first evaluated by the attending physicians and subsequently reviewed by an independent expert panel to confirm their causality and relevance to ulinastatin. Subsequently, logistic regression analysis was employed to identify independent risk factors among the collected variables. Finally, these data were used to establish a risk scoring system (Figure 1).

### 2.3 Dichotomous variables processing

Prior to regression analysis, all variables were assigned numerical values as follows: a history of cardiopulmonary disease was coded as 1, and its absence as 0; ICU admission as 1, and general ward admission as 0; allergy history as 1, and no allergy history as 0; male gender as 1, and female as 0; concomitant medication use as 1, and no concomitant medications as 0. Continuous variables were dichotomized based on clinical relevance: length of hospital stay greater than 7 days was assigned a value of 1 (≤7 days as 0), as most patients in the dataset had a hospital stay in 7 days. According to the prescribing information of ulinastatin (Lv et al., 2020), the recommended initial dose for adult patients with acute pancreatitis or chronic recurrent pancreatitis is 10 × 10^4^ units per administration, diluted in 500 mL of 5% glucose injection or 0.9% sodium chloride injection for intravenous drip infusion over 1–2 h, administered 1–3 times daily. The dose may be gradually reduced as symptoms subside. For acute circulatory failure, the same dosage and dilution are recommended, with the option of slow intravenous injection under certain circumstances. The prescribed daily dose ranges between 10 × 10^4^ and 30 × 10^4^ units (Lyu et al., 2022). Based on the collected data, the average daily dose administered was 30 × 10^4^ units. Therefore, a total daily dose of ≥30 × 10^4^ units was assigned a value of 1, and <30 × 10^4^ units as 0. Duration of medication >1 day was assigned a value of 1, and <1 day as 0. The variable ‘duration of medication >1 day’ was defined as the administration of ulinastatin on more than one calendar day, irrespective of the total infusion hours.

### 2.4 ROC curve

ROC curve analysis was constructed to evaluate the discriminatory performance of the predictive model by plotting the true positive rate (sensitivity, true positive rate, TPR) against the false positive rate (1 – specificity, false positive rate, FPR) across all classification thresholds (Nahm, 2022; Lai et al., 2025). The ROC curve is formed with the FPR as the abscissa (x-axis) and the TPR as the ordinate (y-axis), wherein the coordinates (FPR, TPR) traversed across every conceivable threshold are joined to constitute the complete curve (Zhou et al., 2024). The overall performance was quantified by calculating the AUC, with an AUC of 1.0 indicating perfect discrimination and lower than 0.5 representing no discriminative capacity. The optimal cutoff value was identified based on the point on the curve closest to the top-left corner, maximizing both sensitivity and specificity.

### 2.5 Risk scoring system

A risk scoring system was developed to transform the multivariable logistic regression model into a simple (Chen et al., 2023), clinically applicable tool for predicting cardiopulmonary adverse outcomes. Initially, independent risk factors were identified through multivariable logistic regression, retaining variables with a significance level of p < 0.05. Each factor’s regression coefficient (β) was used to determine its relative weight (Lee et al., 2022). To construct the score, the β coefficient of each significant predictor was divided by a reference value (often the smallest β in the model), and rounded to the nearest integer to assign point values. For instance, a factor with β = 0.70 assigned 1 point, while another with β = 1.40 received 2 points. The points for all predictors were summed to calculate a total risk score for each patient. This score was then categorized into risk strata based on the observed incidence of the outcome in each score range. The total risk scores were then used to generate the ROC curve using SPSS software (version 25.0) (Wu et al., 2021). The discriminatory power of the risk scoring system was evaluated by comparing the area under the curve (AUC) with that of individual risk factors. Finally, risk stratification into high- and low-risk groups was performed to support clinical decision-making and guide intervention strategies.

### 2.6 Statistical analysis

All statistical analyses were performed using IBM SPSS Statistics 25.0 and Microsoft Excel. Categorical variables were compared using the χ^2^ test or Fisher’s exact test, as appropriate. Continuous variables were compared using the Mann-Whitney U test or t-test based on their distribution. Univariate and multivariate logistic regression analyses were employed to calculate ORs and 95% CIs for identifying independent risk factors. A ROC curve was generated to evaluate the predictive performance of both the final multivariate model and the derived risk scoring system. The AUC was compared between the model and individual predictors. A two-sided p-value <0.05 was considered statistically significant.

## 3 Results

### 3.1 Clinical characteristics and outcomes of patients treated with ulinastatin

The analysis included a total of 11,218 patients treated with ulinastatin, after the exclusion of 34 individuals due to incomplete baseline information. Among this cohort, 152 patients (1.35%) experienced cardiopulmonary adverse outcomes (11,066 (98.65%) did not), establishing a clear patient safety endpoint for further investigation. The total dosage of ulinastatin administered ranged from 2 × 10^4^ to 1.30 × 10^4^ U, with a mean dose of 30.27 × 10^4^ U. Univariate analysis aimed to identify clinical practices and patient profiles associated with this risk. It revealed significant associations with several identifiable factors. Specifically, a higher incidence of adverse outcomes was observed in patients receiving a total ulinastatin dosage of ≤30 × 10^4^ U (93.42% vs. 69.00% in the general ward group; p < 0.001), those with concomitant medication use (61.84% vs. 40.59%; p < 0.001), ICU patients (77.63% vs. 62.10%; p < 0.001), and those with medication duration >1 day (69.10% vs. 40.20%; p < 0.001). In contrast, no statistically significant differences were found in sex (male: 65.8% vs. 65.10%; p = 0.850), allergy history (7.24% vs. 5.02%; p = 0.217), history of cardiopulmonary diseases (57.24% vs. 53.09%; p = 0.309), or length of hospital stay (>7 days: 71.71% vs. 67.24%; p = 0.244) between the two groups. The detailed distribution of all baseline characteristics is presented in Table 2.

**TABLE 2:** Comparison of general clinical data among 11218 patients administered ulinastatin.

Variables	With cardiopulmonary adverse outcomes (N = 152)	Without cardiopulmonary adverse outcomes (N = 11066)	p-value	x2
Ulinastatin dosage/10^4^ U	​	​	<0.001	81.975
≤30	142 (93.42%)	7635 (69.00%)	​	​
>30	10 (6.58%)	3431 (31.00%)	​	​
Sex	​	​	0.850	0.036
Male	100 (65.8%)	7199 (65.10%)	​	​
Female	52 (34.2%)	3867 (34.90%)	​	​
Allergy history	​	​	0.217	1.544
Yes	11 (7.24%)	555 (5.02%)	​	​
No	141 (92.76%)	10511 (94.98%)	​	​
Combination medication	​	​	<0.001	28.013
Yes	94 (61.84%)	4492 (40.59%)	​	​
No	58 (38.16%)	6574 (59.41%)	​	​
ICU patients	​	​	<0.001	15.401
Yes	118 (77.63%)	6872 (62.10%)	​	​
No	34 (22.37%)	4194 (37.90%)	​	​
History of cardiopulmonary diseases	​	​	0.309	1.035
Yes	87 (57.24%)	5875 (53.09%)	​	​
No	65 (42.76%)	5191 (46.91%)	​	​
Duration of medication/Day(s)	​	​	<0.001	51.803
≤1	47 (30.90%)	6616 (59.80%)	​	​
>1	105 (69.10%)	4450 (40.20%)	​	​
Length of hospital stay/Day(s)	​	​	0.244	1.361
≤7	43 (28.29%)	3625 (32.76%)	​	​
>7	109 (71.71%)	7441 (67.24%)	​	​

Data are $n\left(\%\right)$ unless otherwise stated.

Chi-square test and univariable logistic regression analysis were performed on the above data. A value of p (Two-tailed) < 0.05 was considered statistically significant, indicating factors influencing the occurrence of cardiopulmonary adverse outcomes.

To further delineate the clinical burden of these events, we categorized the specific types of cardiopulmonary adverse outcomes among the 152 affected patients. Among patients who experienced cardiopulmonary adverse outcomes, the top five conditions in the ICU setting were: severe pneumonia (39 cases, 33.05%), respiratory and circulatory failure (36 cases, 30.51%), respiratory and cardiac arrest (25 cases, 21.19%), cardiogenic shock and heart failure (each 10 cases, 8.47%), and myocardial infarction (9 cases, 7.63%). In the general ward group, the most frequent adverse outcomes were respiratory and circulatory failure (15 cases, 44.12%), severe pneumonia (10 cases, 29.41%), respiratory and cardiac arrest (9 cases, 26.47%), heart failure (3 cases, 8.82%), and myocardial infarction (2 cases, 5.88%). A complete breakdown of adverse outcome types is provided in Table 3, highlighting the severe nature of the events this study aims to help prevent.

**TABLE 3:** Analysis of cardiopulmonary adverse outcomes in 152 cases after ulinastatin administration.

Variables	ICU patients (N = 153)	General patients (N = 46)	Total (N = 199)
Myocardial infarction	9 (7.63%)	2 (5.88%)	11 (7.24%)
Cardiogenic shock	10 (8.47%)	0 (0.00%)	10 (6.58%)
Ventricular fibrillation	3 (2.54%)	1 (2.94%)	4 (2.63%)
Severe pneumonia	39 (33.05%)	10 (29.41%)	49 (32.24%)
Heart failure	10 (8.47%)	3 (8.82%)	13 (8.55%)
Respiratory and cardiac arrest	25 (21.19%)	9 (26.47%)	34 (22.37%)
Pulmonary infection	4 (3.39%)	1 (2.94%)	5 (3.29%)
Postcardiotomy low cardiac output syndrome	1 (0.85%)	2 (5.88%)	3 (1.97%)
Respiratory and circulatory failure	36 (30.51%)	15 (44.12%)	51 (33.55%)
Lung malignancy	0 (0.00%)	1 (2.94%)	1 (0.66%)
COPD	5 (4.24%)	2 (5.88%)	7 (4.61%)
Coronary heart disease	7 (5.893%)	0 (0.00%)	7 (4.61%)
Hypertension	4 (3.39%)	0 (0.00%)	4 (2.63%)

Data are $n\left(\%\right)$ unless otherwise stated.

### 3.2 Independent risk factors for cardiopulmonary adverse outcomes after ulinastatin treatment

To identify independent and potentially modifiable predictors for preventive health strategies, all variables significant in univariate analysis were entered into a multivariable logistic regression model. These variables assessed included ulinastatin dosage, duration of medication/day(s), combination medication, ICU patients, history of cardiopulmonary diseases, length of hospital stay/day(s), sex, allergy history. The analysis revealed several independent predictors of cardiopulmonary adverse outcomes in patients receiving ulinastatin. A total ulinastatin dosage (low-dose) of ≤30 × 10^4^ U was the strongest medication-related risk factor, associated with a more than five-fold increase in odds (OR = 5.570, 95% CI: 3.670–8.454, p < 0.001).

Admission to the ICU itself was the strongest overall predictor (OR = 3.737, 95% CI: 2.487–5.615, p < 0.001), highlighting a critical high-risk patient population. Prolonged medication duration (>1 day) was an independent risk factor (OR = 2.165, 95% CI: 1.480–3.166, p < 0.001). Concomitant medication use also significantly elevated the risk (OR = 2.088, 95% CI: 1.414–3.083, p < 0.001). In contrast, history of cardiopulmonary disease (OR = 0.962, 95% CI: 0.670–1.382, p = 0.846), length of hospital stay (OR = 0.780, 95% CI: 0.536–1.135, p = 0.194), sex (OR = 0.971, 95% CI: 0.689–1.367, p = 0.865), as well as allergy history (OR = 0.955, 95% CI: 0.505–1.805, p = 0.886) did not demonstrate statistically significant associations in the multivariable analysis (Detailed results are presented in Table 4). These results indicate that lower ulinastatin dosage (≤30 × 10^4^ U), longer medication duration, concomitant medication use, and ICU admission are significant independent risk factors for cardiopulmonary adverse outcomes in patients treated with ulinastatin.

**TABLE 4:** Correlation between ulinastatin use and cardiopulmonary adverse outcomes in 11218 patients.

Variables	p-value	OR	95% CI
Ulinastatin dosage/10^4^ U	<0.001	5.570	3.670,8.454
Duration of medication/Day(s)	<0.001	2.165	1.480,3.166
Combination medication	<0.001	2.088	1.414,3.083
ICU patients	<0.001	3.737	2.487,5.615
History of cardiopulmonary diseases	0.846	0.962	3.670,8.454
Length of hospital stay/Day(s)	0.194	0.780	0.536,1.135
Sex	0.865	0.971	0.689,1.367
Allergy history	0.886	0.955	0.505,1.805

Data are $n\left(\%\right)$ unless otherwise stated. OR = odds ratio. Variables with statistical significance in the univariate analysis were included in the multivariate logistic regression model to identify independent risk factors and construct a predictive model.

### 3.3 Development of a clinically actionable risk scoring tool

To translate the identified risk factors into a practical tool for preventive health screening, we developed a simple risk scoring system based on the regression coefficients (β) from the multivariable model. The goal was to create an intuitive score that could be easily calculated at the point of care to guide clinical behavior. The smallest β-value (0.736, for concomitant medication use) was used as the reference to standardize point assignments. The relative weight for each variable was calculated by dividing its β-coefficient by this reference value, followed by rounding to the nearest integer to assign practical point scores. The resulting points-based tool integrates the four independent risk factors as follows: ulinastatin dosage <30 × 10^4^ U (β = 1.717, standardized value = 2.33, assigned 2 points) and ICU admission (β = 1.318, standardized value = 1.79, assigned 2 points) were the strongest predictors. Medication duration >1 day (β = 0.772, standardized value = 1.05, assigned 1 point) and concomitant medication use (β = 0.736, assigned 1 point) were also included in the scoring system. This yields a total risk score ranging from 0 to 6. The detailed derivation is shown in Table 5. This risk scoring system transforms complex statistical predictions into a readily applicable clinical tool. By allowing for the immediate stratification of patients into distinct risk categories based on routinely available data, it facilitates proactive, evidence-based decision-making. This directly supports the promotion of safer medication practices by enabling clinicians to identify high-risk patients prior to or during treatment, thereby targeting monitoring resources and guiding preventive strategies.

**TABLE 5:** Risk scoring system for cardiopulmonary adverse outcomes in patients administered ulinastatin.

Variables	β	β/0.736	Score
Ulinastatin dosage/10^4^ U	1.717	2.33	​
<30	​	​	2
≥30	​	​	0
Duration of medication/Day(s)	0.772	1.04	​
≤1	​	​	0
>1	​	​	1
Combination medication	0.736	1.00	​
Yes	​	​	1
No	​	​	0
ICU patients	1.318	1.79	​
Yes	​	​	2
No	​	​	0

The regression coefficient (β) of each factor serves as the basis for determining its relative weight. In the process of constructing the score, the β-coefficient of each significant predictor is divided by the reference value within the model, specifically the value corresponding to the predictor with the smallest β-value. Subsequently, the resulting quotient is rounded to the nearest integer, thereby assigning a score.

### 3.4 Performance of the risk assessment system using ROC curve analysis

**FIGURE 2:**
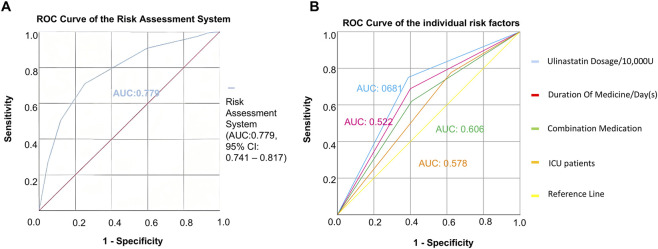
Predictive performance of the risk assessment model. **(A)** ROC curve for the composite risk score. The area AUC was 0.779 (95% CI: 0.741–0.817). **(B)** Comparison of the ROC curves between the composite risk score and individual clinical predictors, demonstrating the superior discriminatory power of the integrated model.

The predictive accuracy of the developed risk scoring system was evaluated using ROC curve analysis. Based on the scoring criteria established from multivariable regression, each of the 11,218 patients included in the study was assigned a total risk score ranging from 0 to 6. These scores were used as the test variable, with the actual occurrence of cardiopulmonary adverse outcomes serving as the state variable. The resulting ROC curve (Figure 2A) demonstrated favorable discriminatory performance, with an AUC of 0.779 (95% CI: 0.741–0.817). This indicates that the risk scoring system has good overall accuracy in distinguishing between patients who experienced cardiopulmonary adverse outcomes and those who did not. An AUC value of 0.779 suggests that the model possesses clinically useful predictive ability, significantly exceeding random chance (AUC = 0.5) (Thomas et al., 2015). The narrow confidence interval further supports the robustness of this estimate. These findings confirm that the risk assessment system, incorporating four readily available clinical variables, serves as a reliable tool for stratifying patients according to their risk of developing cardiopulmonary adverse outcomes following ulinastatin administration.

### 3.5 Predictive performance of the risk assessment system for cardiopulmonary adverse outcomes

To assess its prognostic utility, the performance of the newly developed risk assessment system was quantitatively evaluated against individual clinical predictors using ROC analysis. The composite risk score demonstrated significantly superior predictive accuracy, yielding an AUC of 0.779 (95% CI: 0.741–0.817). In contrast, the predictive capacities of the individual risk factors were considerably lower: ulinastatin dosage <30 × 10^4^ U (AUC = 0.681, 95% CI: 0.640–0.721), combination medication use (AUC = 0.606, 95% CI: 0.561–0.651), ICU admission (AUC = 0.578, 95% CI: 0.535–0.620), and medication duration >1 day (AUC = 0.522, 95% CI: 0.477–0.568) (Table 6; Figure 2B). To transform the risk score into an actionable tool for clinical practice, an optimal cutoff value of ≥3.5 was established using Youden’s index, providing the optimal balance between sensitivity and specificity for identifying high-risk patients (Tang et al., 2024). As the total score is an integer, this cut-off is operationally applied such that a score of 0–3 is classified as low-risk, and a score of ≥4 is classified as high-risk. This threshold enables reliable stratification of patients into distinct risk categories, facilitating early identification of those who would benefit most from intensified monitoring and preventive care strategies. These findings affirm that integrating multiple predictors into a simple composite score provides a more robust foundation for clinical decision-making and resource allocation than relying on any single clinical indicator.

**TABLE 6:** Evaluation of the risk assessment system for cardiopulmonary adverse outcomes.

Factors	AUC	95% CI
Risk assessment system	0.779	0.741,0.817
ICU	0.578	0.535,0.620
Combination medication	0.606	0.561,0.651
Medication dosage <30 × 10^4^ U	0.681	0.640,0.721
Duration of medication >1 Day	0.522	0.477,0.568

The analytical assessment system was compared with individual risk factors. AUC, Area Under the ROC, curve.

## 4 Discussion

Medication safety is a pivotal public health issue, and the safe application of immunomodulatory agents like ulinastatin in complex clinical settings represents a significant challenge and opportunity for health promotion. While ulinastatin, a broad-spectrum protease inhibitor, exerts its anti-inflammatory and immunomodulatory effects primarily by inhibiting excessive proteolytic activity (e.g., from trypsin and elastase), which in turn mitigates subsequent inflammatory cascade and organ injury. (Inoue et al., 2005; Xu et al., 2013), our findings underscore that its benefits are contingent upon appropriate clinical deployment. The identification of a low daily dose (<300,000 U) as the strongest modifiable risk factor for cardiopulmonary adverse outcomes (OR = 5.57) is a critical insight. This suggests that, in vulnerable populations, subtherapeutic dosing fails to provide adequate immunomodulatory coverage, potentially allowing uncontrolled inflammation to propagate organ injury. This is consistent with the drug’s short half-life (∼40 min) and the dose-dependent anti-inflammatory effects observed in preclinical models of lung injury (Xu et al., 2008). In clinical practice, it is plausible that clinicians tended to administer higher doses of ulinastatin to patients perceived as having more severe conditions or higher risk, while lower doses were used for patients with less severe presentations. This prescribing pattern could create a spurious association where the administered dose serves as a proxy for underlying disease severity. To address this concern, we analyzed dose as a continuous variable and observed a trend supporting the categorical findings. As the prescribing information for ulinastatin recommends a daily dose range of 10 × 10^4^ to 30 × 10^4^ units, we stratified patients by these dosage levels (e.g.,.above 10 × 10^4^, 20 × 10^4^, 30 × 10^4^), and found a dose of 30 × 10^4^ appeared to represent a threshold for a significantly lower risk of cardiopulmonary adverse outcomes. While these analytical approaches mitigate the concern to some extent, we fully acknowledge that residual confounding by unmeasured severity factors cannot be entirely ruled out. This highlights the need for future prospective, ideally randomized studies to definitively establish the causal effect of ulinastatin dosing. Beyond dosage, the other identified risk factors, ICU admission, prolonged therapy, and concomitant medications, collectively paint a picture of the complex, high-risk patient in whom clinical decision-making is most challenging. ICU admission serves as a proxy for severe immune dysregulation. Prolonged therapy often indicates persistent or difficult-to-control inflammation, and polypharmacy increases the risk of unforeseen drug interactions and compounded toxicities. Our study moves beyond merely identifying these risks by integrating them into a practical risk stratification tool. The robust performance of this tool (AUC = 0.779), which significantly outperformed any single predictor, validates the necessity of a holistic assessment over relying on isolated clinical cues.

The primary public health implication of our study lies in the translation of this risk score into actionable strategies for promoting safer medication use. A score at or above the cutoff of 4 effectively identifies patients who warrant a pre-emptive, intensified management protocol. This enables a shift from a reactive to a preventive model of care—a core principle of health promotion. For instance, high-risk patients could be flagged in the electronic health record (EHR) for automatic clinical decision support, triggering interventions such as first, protocol-driven dose optimization to avoid underdosing; second, enhanced monitoring (e.g., serial ECGs, biomarker assessment); and third, structured medication review by a clinical pharmacist to manage polypharmacy risks. This approach aligns with established health behavior models by providing a clear, easy-to-use tool that can guide and improve clinician behavior at the point of care.

We further propose that this risk model be integrated into hospital-based safety initiatives and professional training programs. Educating clinicians on these specific, modifiable risk factors and equipping them with a simple scoring system can foster a culture of safety and shared decision-making. This directly addresses the ‘how’ of health promotion in a clinical context, moving from knowledge to tangible practice.

In conclusion, this study transcends the conventional analysis of drug safety by developing and validating a practical tool for risk stratification. By pinpointing modifiable clinical practices and providing a means to identify at-risk individuals, our work offers a concrete strategy to enhance patient safety protocols. It provides a framework for optimizing clinical behavior, guiding resource allocation, and ultimately promoting healthier outcomes for patients receiving ulinastatin, thereby contributing meaningfully to the fields of medication safety and public health.

## 5 Conclusion

This study developed a validated risk prediction model for cardiopulmonary adverse events in patients receiving ulinastatin. The findings highlight that insufficient dosing is a critical, modifiable risk factor and provide a practical tool for preemptive risk stratification. Implementing this score can guide clinical decision-making, optimize medication safety, and promote healthier outcomes in vulnerable populations.

## Data Availability

The raw data supporting the conclusions of this article will be made available by the authors, without undue reservation.
